# Naltrexone Restores Impaired Transient Receptor Potential Melastatin 3 Ion Channel Function in Natural Killer Cells From Myalgic Encephalomyelitis/Chronic Fatigue Syndrome Patients

**DOI:** 10.3389/fimmu.2019.02545

**Published:** 2019-10-31

**Authors:** Helene Cabanas, Katsuhiko Muraki, Donald Staines, Sonya Marshall-Gradisnik

**Affiliations:** ^1^School of Medical Science, Griffith University, Gold Coast, QLD, Australia; ^2^The National Centre for Neuroimmunology and Emerging Diseases, Menzies Health Institute Queensland, Griffith University, Gold Coast, QLD, Australia; ^3^Consortium Health International for Myalgic Encephalomyelitis, National Centre for Neuroimmunology and Emerging Diseases, Griffith University, Gold Coast, QLD, Australia; ^4^Laboratory of Cellular Pharmacology, School of Pharmacy, Aichi-Gakuin University, Nagoya, Japan

**Keywords:** transient receptor potential melastatin 3, naltrexone, calcium, opioid receptor, myalgic encephalomyelitis/chronic fatigue syndrome, natural killer cells, whole-cell patch clamp electrophysiology

## Abstract

Myalgic Encephalomyelitis/Chronic Fatigue Syndrome (ME/CFS) is a seriously long-term and debilitating illness of unknown cause hallmarked by chronic pain and fatigue, memory and concentration impairment, and inflammation. ME/CFS hypothesis involves impaired Transient receptor potential melastatin 3 (TRPM3) ion channel function, affecting calcium signaling and Natural killer (NK) cell functions. Currently, substances called opioids, agonists of mu (μ)-opioid receptors (μOR), are the strongest painkillers clinically available for people suffering from strong or long-lasting pain characteristic of ME/CFS. μOR have been reported to specifically inhibit TRPM3 and to be expressed in immune cells where they play an immunomodulatory and immunosuppressive role. Naltrexone hydrochloride (NTX) acts as an antagonist to the μOR thus negating the inhibitory function of this opioid receptor on TRPM3. Therefore, understanding the mechanism of action for NTX in regulating and modulating TRPM3 channel function in NK cells will provide important information for the development of effective therapeutic interventions for ME/CFS. Whole-cell patch-clamp technique was used to measure TRPM3 activity in Interleukin-2 (IL-2) stimulated and NTX-treated NK cells for 24 h on eight ME/CFS patients and 8 age- and sex-matched healthy controls, after modulation with a TRPM3-agonist, pregnenolone sulfate (PregS), NTX and a TRPM3-antagonist, ononetin. We confirmed impaired TRPM3 function in ME/CFS patients through electrophysiological investigations in IL-2 stimulated NK cells after modulation with PregS and ononetin. Importantly, TRPM3 channel activity was restored in IL-2 stimulated NK cells isolated from ME/CFS patients after incubation for 24 h with NTX. Moreover, we demonstrated that NTX does not act as an agonist by directly coupling on the TRPM3 ion channel gating. The opioid antagonist NTX has the potential to negate the inhibitory function of opioid receptors on TRPM3 in NK cells from ME/CFS patients, resulting in calcium signals remodeling, which will in turn affect cell functions, supporting the hypothesis that NTX may have potential for use as a treatment for ME/CFS. Our results demonstrate, for the first time, and based on novel patch clamp electrophysiology, potential pharmaco-therapeutic interventions in ME/CFS.

## Introduction

Myalgic Encephalomyelitis/Chronic Fatigue Syndrome (ME/CFS) is a severe systemic, acquired condition of unknown cause characterized by persistent or recurrent incapacitating fatigue accompanied by a range of multi-system manifestations (1). Currently, there are no confirmatory diagnostic tests or accepted specific treatments. Diagnosis is currently based on the Canadian Consensus Criteria (CCC, 2003) and more recently on the International Case Criteria (ICC, 2011), which identifies symptoms of post-exertional malaise, fatigue unrelieved by rest, headache, joint and muscle pain, memory and concentration impairment, sore throat, and lymph gland swelling as components of the illness (2). Additionally, ME/CFS exhibits neuro-cognitive, autonomic, cardiovascular, neuro-endocrine, and immune manifestations.

Although the etiology of ME/CFS remains elusive, immune dysfunction and abnormalities in natural killer (NK) cell functions are the most consistent laboratory immunological features (3–18). NK cells are lymphocytes of the innate immune system that control pathogens and tumor cells by limiting their spread and subsequent tissue damage under the stimulation of the endogenous interleukin-2 (IL-2) and interleukin-12 (IL-12), in addition to immune cell activation and cytokine production (19). While there is an inconsistency in some immunological reports, differences in NK cell phenotypes and significantly reduced peripheral NK cell numbers resulting in significant reduction in NK cell cytotoxicity, have been reported in ME/CFS and implicated in disease severity (3–18).

Importantly, NK cells require calcium (Ca^2+^) for efficient stimulation and functions including NK cell cytotoxicity (20–23). Intracellular Ca^2+^ concentration ([Ca^2+^]_i_) is tightly regulated by numerous actors including selective and non-selective channels, pumps, and exchangers located on the plasma membrane and/or on organelles. Disturbance of this tightly regulated homeostatic system leads to disorders of Ca^2+^ metabolism and immune cell functions playing a pivotal role in the pathophysiology of several diseases and immunodeficiencies (24).

One major contributor to Ca^2+^ signaling is the large and diverse family of Transient Receptor Potential (TRP) non-selective cation channels, which function as polymodal cellular sensors involved in the fine-tuning of many biological processes in both excitable and non-excitable cells (25). The mammalian TRP melastatin (TRPM) subfamily is the largest, consisting of eight members broadly expressed in a variety of cells and tissues, such as sensory ganglia, the central nervous system (CNS), pancreatic beta cells, immune cells, cardiovascular system and renal system, and are critical for sensory and motor physiology (26). Specifically, TRPM member 3 (TRPM3) is a Ca^2+^-permeable nonselective cation channel activated by a vast array of stimuli in the environment, ranging from temperature, natural chemicals, and toxins or synthetic compounds, including the endogenous neurosteroid pregnenolone sulfate (PregS), and the L-type voltage-gated Ca^2+^ channel inhibitor, nifedipine, to mechanical stimuli. TRPM3 channels also respond to endogenous agents and messengers produced during tissue injury and inflammation (27). Importantly, TRPM3 has been previously identified as a thermosensitive and nociceptor channel implicated in the detection of acute heat sensing and inflammatory heat hyperalgesia as well as in pain transmission in the CNS (27). TRPM3 is therefore involved in several pain-related pathological conditions/modalities, including inflammatory or neuropathic pain, and thus is identified as a potential target for analgesic treatments. Moreover, dysregulation of thermoregulatory responses has been reported in ME/CFS patients and generalized pain in the absence of overt tissue damage is a characteristic of ME/CFS suggesting potential CNS impairments (1).

Our previous studies have reported a loss of TRPM3 ion channel to be associated with ME/CFS (28, 29). A previous genomic study suggested the importance of TRPM3 in the pathophysiology of ME/CFS and identified five single nucleotide polymorphisms (SNPs) (rs6560200, rs1106948, rs12350232, rs11142822, rs1891301) in *TRPM3* genes in ME/CFS patients (30). Significant reduction in TRPM3 surface expression and Ca^2+^ mobilization in immune cells were subsequently reported in ME/CFS patients (31, 32). Recently, novel electrophysiological investigations used whole-cell patch clamp techniques to report a significant reduction in TRPM3 ion channel activity after PregS and nifedipine stimulation in NK cells from ME/CFS patients (28, 29). Moreover, ionic currents in ME/CFS patients were resistant to ononetin in the presence of PregS and nifedipine. Consequently, dysregulation of TRPM3 function in ME/CFS patients, affecting [Ca^2+^]_i_ and Ca^2+^ signaling has significant implications for NK cell regulatory machinery and functions, and represents a novel and attractive therapeutic target of ME/CFS pathology.

There are few treatments available for people suffering from severe or long-lasting pain characteristic of ME/CFS. Currently, substances called opioids, agonists of mu (μ)-opioid receptors (μOR), are the strongest painkillers clinically available (33). Opioids mediate their effects by interacting with molecules that belong to a group of receptor proteins called G-protein coupled receptors (GPCRs). These opioid receptors are widely distributed in the CNS with the role of detecting and transmitting pain signals (33). It was poorly understood how activation of opioid receptors reduces the activity of pain-sensing nerve cells, however recent literature suggests that activation of GPCRs can affect TRPM3 channels and in turn decrease the flow of Ca^2+^ ions through the pore (33–35). GPCRs interact with G-proteins that, when activated by the receptor, release the Gβγ dimers from Gα subunits of the G_i/o_ subfamily. Inhibition of TRPM3 activity by stimulation of GPCRs (in particular μORs) is mediated through a direct binding of the G_βγ_ subunit to the ion channel (34). These recent findings show that drugs already used in the treatment of pain can indirectly alter TRPM3 function significantly (33).

Naltrexone hydrochloride (NTX) is a long-lasting opioid antagonist used commonly in the treatment of opioid and alcohol dependence (36). NTX specifically inhibits μORs and, to a lesser extent, the delta (δ)-opioid receptors (δOR), thus negating the inhibiting effects of opioid receptors agonists (37, 38). A recent investigation demonstrated that naloxone, a rapid response alternative to naltrexone, did not have a direct effect on TRPM3-dependent Ca^2+^ signals in mouse dorsal root ganglion neurons (33). However, when co-applied with DAMGO, a highly selective μOR agonist, naloxone prevented the action of DAMGO completely, indicating a possible role for naloxone in influencing TRPM3 signaling. Interestingly, TRPM3 activation by nifedipine and PregS was also inhibited by μOR activation confirming that TRPM3 inhibition is an important consequence of peripheral μOR activation (33, 35). Moreover, it has been suggested that treatment with low-dose naltrexone (LDN) can act as an immunomodulator and may be beneficial for a range of inflammatory conditions, including Crohn's disease, multiple sclerosis, and fibromyalgia (39–41). Previous studies also report therapeutic effects of LDN in treatment for cancers including B cell lymphoma and pancreatic cancer, as well as chronic pain syndromes, malignancies and mental health disorders by reducing pain, fatigue, sleep disturbances, headaches and gastrointestinal conditions (42).

As ME/CFS is potentially a TRP ion channel disorder resulting from impaired TRPM3 ion channel function (28–32), understanding the mechanism(s) involved in regulating and modulating ion channel function will provide potentially important information and lead to novel targets for the development of effective therapeutic interventions. In addition, doses of 3–5 mg per day of LDN have been used to treat a variety of diseases, including ME/CFS (42, 43). However, there is no literature confirming the efficacy of NTX for ME/CFS patients, hence the usefulness of this drug in this condition is yet to be determined. Additionally, the mechanisms of action for NTX in immune cells and in ME/CFS pathology require further investigation. As TRPM3 ion channel function is impaired in NK cells from ME/CFS patients (28, 29), in the present study we sought to investigate the ability of NTX to restore TRPM3 ion channel function in ME/CFS using whole cell patch-clamp techniques. Restoration of TRPM3 function is required to promote Ca^2+^ signal remodeling in NK cells. Our results indicate that NTX restores TRPM3-like ionic currents in IL-2 stimulated NK cells from ME/CFS patients following 24 h incubation. Our findings also indicate that NTX does not affect TRPM3-dependent Ca^2+^ signals when directly applied on both IL-2 stimulated NK cells from non-fatigued controls and ME/CFS patients, suggesting that NTX does not act as an agonist by directly coupling on the TRPM3 ion channel gating mechanism. In conclusion, our novel findings indicate that NTX has the potential to restore the TRPM3 channel activity in ME/CFS patients resulting in Ca^2+^ signal remodeling, which will in turn affect cell functions, supporting the hypothesis that NTX may have potential for use as a treatment for ME/CFS.

## Materials and Methods

### Participant Recruitment

Eight ME/CFS patients and 8 age- and sex-matched healthy controls (HC) were recruited using the National Center for Neuroimmunology and Emerging Diseases (NCNED) research database between April and June 2019. Participants were aged between 18 and 60 years. All ME/CFS patients had previously received a confirmed medical diagnosis and were screened using a comprehensive questionnaire corresponding with the Centers for Disease Control and Prevention (CDC), CCC, and ICC case definitions. All eight ME/CFS patients were defined by the CCC. HC reported no incidence of fatigue and were in good health without evidence of illness. Participants were excluded from this study if they were pregnant or breastfeeding, or reported a previous history of smoking, alcohol abuse or chronic illness (for example, autoimmune diseases, cardiac diseases, and primary psychological disorders) or were morbidly obese (BMI ≥ 30). No participants reported use of opioids or any other pain killers in the preceding 3 months as well as pharmacological agents that directly or indirectly influence TRPM3 or Ca^2+^ signaling. This investigation was approved by the Griffith University Human Research Ethics Committee (HREC/15/QGC/63).

### Peripheral Blood Mononuclear Cell Isolation and Natural Killer Cell Isolation

A total of 85 ml of whole blood was collected in ethylendiaminetetraacetic acid (EDTA) tubes between 8:30 and 10:30 a.m. on the Gold Coast, QLD, Australia. Routine full blood analysis was performed within 4 h of collection for red blood cell count, white blood cell count, and granulocyte cell count.

Samples were provided to the laboratory de-identified using a unique code by an independent blood collector. Peripheral blood mononuclear cells (PBMCs) were isolated from 80 ml of whole blood by centrifugation over a density gradient medium (Ficoll-Paque Premium; GE Healthcare, Uppsala, Sweden) as previously described (3, 44). PBMCs were stained with trypan blue (Invitrogen, Carlsband, CA, USA) to determine cell count and cell viability. PBMCs were adjusted to a final concentration of 5 × 10^7^ cells/ml for NK cell isolation.

NK cells were isolated by immunomagnetic selection using an EasySep Negative Human NK Cell Isolation Kit (Stem Cell Technologies, Vancouver, BC, Canada). NK Cell purification was determined using flow cytometry. NK cells were incubated for 20 min at room temperature in the presence of CD56 FITC (0.25 μg/5 μl) and CD3 PE Cy7 (0.25 μg/20 μl) monoclonal antibodies (BD Bioscience, San Jose, CA, USA) as previously described (32). 7-amino-actinomycin (7-AAD) (2.5 μl/test) (BD Bioscience, San Jose, CA, USA) was used to determine cell viability. Cells were washed and resuspended in 200 μl of stain buffer (BD Bioscience, New Jersey, NJ, USA) and acquired at 10,000 events using the LSRFortessa X-20 (BD Biosciences, San Diego, CA, USA). Using forward and side scatter, the lymphocyte population was gated while acquiring the sample. The NK cell population was then identified as CD3^−^CD56^+^ cells.

### Interleukin-2 Stimulation and Drug Treatment

Freshly isolated NK cells were stimulated with 20 IU/ml of recombinant human IL-2 (specific activity 5 × 10^6^ IU/mg) (Miltenyi Biotech, BG, Germany) in combination or not with 200 μM NTX (Sigma-Aldrich, St. Louise, MO, USA) as previously described (36), at a concentration of 2 × 10^5^ cells/ml and incubated for 24 h at 37°C with 5% CO_2_ in Roswell Park Memorial Institute medium (RPMI)-1640 (Invitrogen Life Technologies, Carlsbad, CA, USA) supplemented with 10% fetal bovine serum (FBS) (Invitrogen Life Technologies, Carlsbad, CA, USA). NTX was freshly resuspended in endotoxin-free water before each experiment.

### Whole Cell Electrophysiology Recording

Electrophysiological recordings were performed with borosilicate glass capillary electrodes with an outside diameter of 1.5 mm and inside diameter of 0.86 mm (Harvard Apparatus, Holliston, MA, USA). Pipette resistance when filled with pipette solution was 8–12 MΩ. The pipettes were mounted on a CV203BU head-stage (Molecular Devices, Sunnyvale, CA, USA) connected to a 3-way coarse manipulator and a micro-manipulator (Narishige, Tokyo, Japan). Electrical signals were amplified and recorded using an Axopatch 200B amplifier and PClamp 10.7 software (Molecular Devices, Sunnyvale, CA, USA). Data were filtered at 5 kHz and sampled digitally at 10 kHz via a Digidata 1440A analog to digital converter (Molecular Devices, Sunnyvale, CA, USA). The voltage-ramp protocol was a step from a holding potential of +10 mV to −90 mV, followed by a 0.1 s ramp to +110 mV, before returning to +10 mV (repeated every 10 s). The liquid junction potential between the pipette and bath solutions (+10 mV) was corrected when data were analyzed. A leak current component was not subtracted from the recorded currents. Electrode was filled with the intracellular pipette solution containing 30 mM CsCl, 2 mM MgCl_2_, 110 mM L-Aspartic acid, 1 mM EGTA, 10 mM HEPES, 4 mM ATP, 0.1 mM GTP, adjusted pH to 7.2 with CsOH and osmolality of 290 mOsm/L with D-mannitol. The pipette solution was filtered using a 0.22 μm membrane filter (Sigma-Aldrich, St. Louise, MO, USA), divided into aliquots and stored at −20°C. Bath solution contained: 130 mM NaCl, 10 mM CsCl, 1 mM MgCl_2_, 1.5 mM CaCl_2_2H_2_O, 10 mM HEPES, adjusted pH to 7.4 with NaOH and osmolality 300 mOsm/L with D-glucose. All reagents were purchased from Sigma-Aldrich, except for ATP and GTP that were purchased from Sapphire Bioscience. TRPM3 currents on non-treated or NTX-treated NK cells were stimulated by adding 100 μM PregS (Tocris Bioscience, Bristol, UK) or 200 μM NTX hydrochloride (Sigma-Aldrich, St. Louise, MO, USA) to the bath solution, whereas PregS- and NTX-induced TRPM3 currents were blocked by adding 10 μM ononetin (Tocris Bioscience, Bristol, UK). Therefore, three different protocols have been performed for each of the 8 ME/CFS patients and 8 age- and sex-matched HC. For each protocol, 4 different recordings were made. Hence, *N* refers to number of participants in each group (ME/CFS patients or HC) and *n*, to number of readings or sample size for each different protocol. All measurements were performed at room temperature. The authors removed the possibility of chloride current involvement in TRPM3 assessment by using L-Aspartic acid in the intracellular pipette solution. Cells which have unstable currents were also excluded from the analysis.

### Statistical Analysis

**Figure 1:**
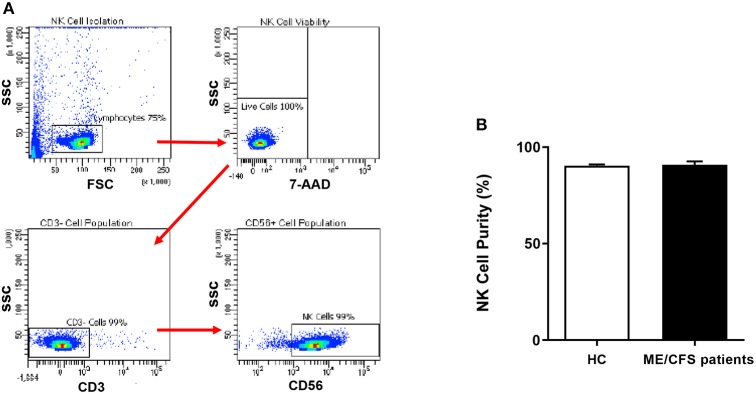
Natural Killer cell purity. **(A)** Gating strategy used to identify NK cells. Representative flow cytometry plots from the PBMCs of one of the study participants. The lymphocytes were live gated during acquisition using the side and forward scatter dot plot display and then single and dead cells were excluded. Furthermore, by using the negative and positive gating strategies, CD3^−^ as well as CD56^+^ lymphocyte populations were identified. **(B)** Bar graphs representing isolated NK cell purity for HC and ME/CFS patients. Data presented as mean ± SEM. HC = 90.34% ± 0.6782 and ME/CFS = 90.9% ± 1.695. 7-AAD, 7-amino-actinomycin; ME/CFS, myalgic encephalomyelitis/chronic fatigue syndrome; FSC, forward scatter; HC, healthy controls; NK cell, natural killer cell; SSC, side scatter.

**Figure 2:**
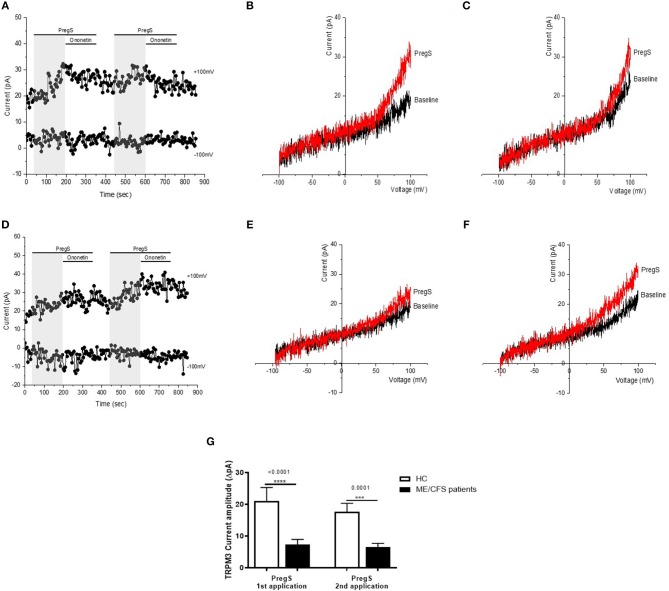
TRPM3 activity after successive applications of PregS. Data were obtained under whole-cell patch clamp conditions. **(A)** A representative time-series of current amplitude at +100 mV and −100 mV showing the effect of successive applications of 100 μM PregS (gray) on ionic currents in IL-2 stimulated NK cells from HC. **(B)** *I*–*V* before and after the first PregS stimulation in a cell corresponding with **(A). (C)** *I*–*V* before and after the second PregS stimulation in a cell corresponding with **(A). (D)** A representative time-series of current amplitude at +100 mV and −100 mV showing the effect of successive applications of 100 μM PregS (gray) on ionic currents in IL-2 stimulated NK cells from ME/CFS patients. **(E)** *I*–*V* before and after the first PregS stimulation in a cell as shown in **(D). (F)** *I*–*V* before and after the second PregS stimulation in a cell as shown in **(D). (G)** Bar graphs representing TRPM3 current amplitude at +100 mV after successive applications of 100 μM PregS in ME/CFS patients (*N* = *8*; *n* = 27 and *n* = 25) compared with HC (*N* = 8; *n* = 31 and *n* = 29). Data are represented as mean ± SEM. ME/CFS, myalgic encephalomyelitis/chronic fatigue syndrome; HC, healthy controls; NK, natural killer; PregS, Pregnenolone sulfate; TRPM3, Transient Receptor Potential Melastatin 3. ***p = 0.0001, ****p < 0.0001.

**Figure 3:**
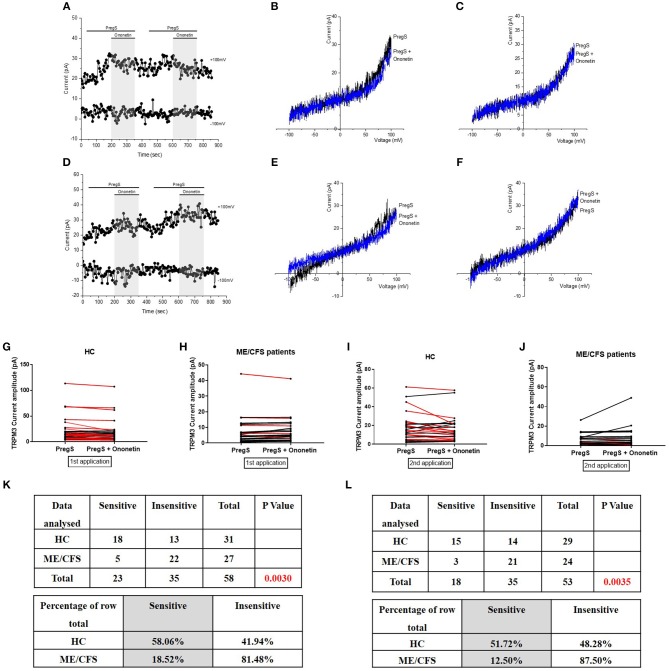
Modulation of PregS- evoked currents with Ononetin. Data were obtained under whole-cell patch clamp conditions. **(A)** A representative time-series of current amplitude at +100 mV and −100 mV showing the effect of 10 μM ononetin (gray) on ionic currents in the presence of 100 μM PregS in IL-2 stimulated NK cells from HC. **(B)** *I*–*V* before and after the first application of ononetin in the presence of PregS in a cell as shown in **(A). (C)** *I*–*V* before and after the second application of ononetin in the presence of PregS in a cell as shown in **(A). (D)** A representative time-series of current amplitude at +100 mV and −100 mV showing the effect of 10 μM ononetin (gray) on ionic currents in the presence of 100 μM PregS in IL-2 stimulated NK cells ME/CFS patients. **(E)** *I*–*V* before and after the first application of ononetin in the presence of PregS in a cell as shown in **(D)**. **(F)** *I*–*V* before and after the second application of ononetin in the presence of PregS in a cell as shown in **(D). (G,H)** Scatter plots representing change of each current amplitude before and after the first application of ononetin in presence of PregS in all NK cells from HC and ME/CFS patients. Each cell represented as red lines had reduction in currents by ononetin. **(I,J)** Scatter plots representing change of each current amplitude before and after the second application of ononetin in presence of PregS in all NK cells from HC and ME/CFS patients. Each cell represented as red lines had reduction in currents by ononetin. **(K)** Table summarizing data for sensitive and insensitive cells to the first application of 10 μM ononetin in presence of PregS in HC (*N* = 8; *n* = 31) compared to ME/CFS patients (*N* = 8; *n* = 27). **(L)** Table summarizing data for sensitive and insensitive cells to the second application of 10 μM ononetin in presence of PregS in HC (*N* = 8; *n* = 29) compared to ME/CFS patients (*N* = 8; *n* = 24). Data are analyzed with Fisher's exact test. ME/CFS, myalgic encephalomyelitis/chronic fatigue syndrome; HC, healthy controls; NK, natural killer; PregS, Pregnenolone sulfate; TRPM3, Transient Receptor Potential Melastatin 3.

**Figure 4:**
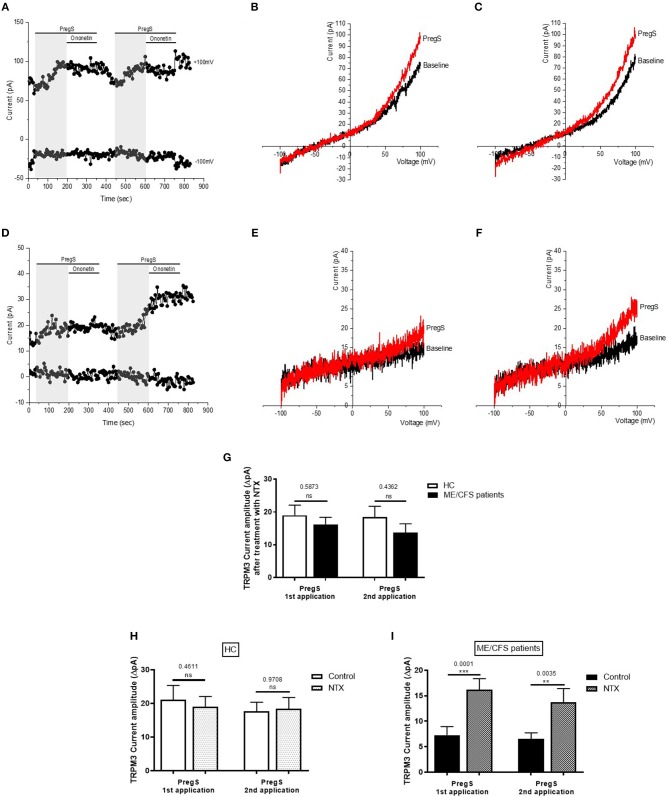
Effects of treatment with NTX on TRPM3 activity after successive applications of PregS. Data were obtained under whole-cell patch clamp conditions. **(A)** A representative time-series of current amplitude at +100 mV and −100 mV showing the effect of successive applications of 100 μM PregS (gray) on ionic currents in IL-2 stimulated NK cells treated with 200 μM NTX for 24 h from HC. **(B)** *I*–*V* before and after the first PregS stimulation in a cell corresponding with **(A). (C)** *I*–*V* before and after the second PregS stimulation in a cell corresponding with **(A). (D)** A representative time-series of current amplitude at +100 mV and −100 mV showing the effect of successive applications of 100 μM PregS (gray) on ionic currents in IL-2 stimulated NK cells treated with 200 μM NTX for 24 h from ME/CFS patients. **(E)** *I*–*V* before and after the first PregS stimulation in a cell as shown in **(D). (F)** *I*–*V* before and after the second PregS stimulation in a cell as shown in **(D). (G)** Bar graphs representing the effect of NTX treatment on TRPM3 current amplitude at +100 mV after successive applications of 100 μM PregS in ME/CFS patients (*N* = *8*; *n* = 22 and *n* = 16) compared with HC (*N* = 8; *n* = 20 and *n* = 19). **(H)** Bar graphs representing TRPM3 current amplitude at +100 mV after successive applications of 100 μM PregS in NK cells treated with 200 μM NTX (*N* = *8*; *n* = 20 and *n* = 19) or NK cells non-treated (*N* = *8*; *n* = 31 and *n* = 29) from HC. The same control has been used as Figure 2G. **(I)** Bar graphs representing TRPM3 current amplitude at +100 mV after successive applications of 100 μM PregS in NK cells treated with 200 μM NTX (*N* = *8*; *n* = 22 and *n* = 16) or NK cells non-treated (*N* = *8*; *n* = 27 and *n* = 25) from ME/CFS patients. The same control has been used as Figure 4G. Data are represented as mean ± SEM. ME/CFS, myalgic encephalomyelitis/chronic fatigue syndrome; HC, healthy controls; NK, natural killer; NTX, naltrexone hydrochloride; PregS, Pregnenolone sulfate; TRPM3, Transient Receptor Potential Melastatin 3. **p = 0.0035, ***p = 0.0001.

**Figure 5:**
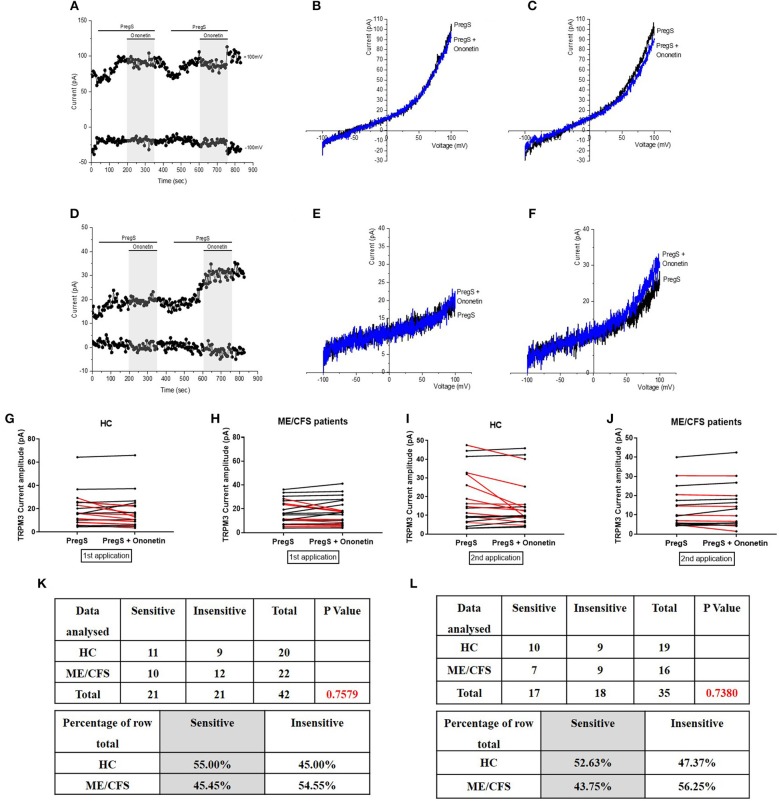
Modulation of PregS- evoked currents with Ononetin after treatment with NTX. Data were obtained under whole-cell patch clamp conditions. **(A)** A representative time-series of current amplitude at +100 mV and −100 mV showing the effect of 10 μM ononetin (gray) on ionic currents in the presence of 100 μM PregS in IL-2 stimulated NK cells treated with 200 μM NTX from HC. **(B)** *I*–*V* before and after the first application of ononetin in the presence of PregS in a cell as shown in **(A). (C)** *I*–*V* before and after the second application of ononetin in the presence of PregS in a cell as shown in **(A). (D)** A representative time-series of current amplitude at +100 mV and −100 mV showing the effect of 10 μM ononetin (gray) on ionic currents in the presence of 100 μM PregS in IL-2 stimulated NK cells treated with 200 μM NTX ME/CFS patients. **(E)** *I*–*V* before and after the first application of ononetin in the presence of PregS in a cell as shown in **(D)**. **(F)** *I*–*V* before and after the second application of ononetin in the presence of PregS in a cell as shown in **(D). (G,H)** Scatter plots representing change of each current amplitude before and after the first application of ononetin in presence of PregS in all NK cells treated with 200 μM NTX from HC and ME/CFS patients. Each cell represented as red lines had reduction in currents by ononetin. **(I,J)** Scatter plots representing change of each current amplitude before and after the second application of ononetin in presence of PregS in all NK cells treated with 200 μM NTX from HC and ME/CFS patients. Each cell represented as red lines had reduction in currents by ononetin. **(K)** Table summarizing data for sensitive and insensitive cells treated with 200 μM NTX to the first application of 10 μM ononetin in presence of PregS in HC (*N* = 8; *n* = 20) compared to ME/CFS patients (*N* = 8; *n* = 22). **(L)** Table summarizing data for sensitive and insensitive cells treated with 200 μM NTX to the second application of 10 μM ononetin in presence of PregS in HC (*N* = 8; *n* = 19) compared to ME/CFS patients (*N* = 8; *n* = 16). Data are analyzed with Fisher's exact test. ME/CFS, myalgic encephalomyelitis/chronic fatigue syndrome; HC, healthy controls; NK, natural killer; NTX, naltrexone hydrochloride; PregS, Pregnenolone sulfate; TRPM3, Transient Receptor Potential Melastatin 3.

**Figure 6:**
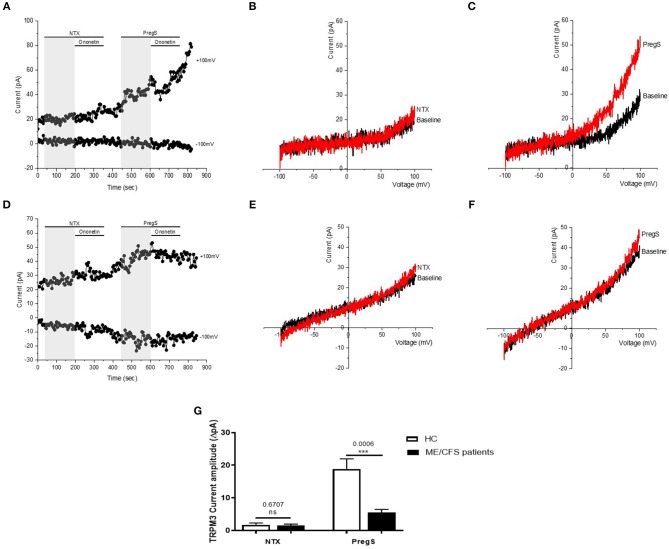
TRPM3 activity after successive applications of NTX and PregS. Data were obtained under whole-cell patch clamp conditions. **(A)** A representative time-series of current amplitude at +100 mV and −100 mV showing the effect of 100 μM PregS and 200 μM NTX (gray) on ionic currents in IL-2 stimulated NK cells from HC. **(B)** *I*–*V* before and after NTX stimulation in a cell corresponding with **(A). (C)** *I*–*V* before and after PregS stimulation in a cell corresponding with **(A). (D)** A representative time-series of current amplitude at +100 mV and −100 mV showing the effect of 100 μM PregS and 200 μM NTX (gray) on ionic currents in IL-2 stimulated NK cells from ME/CFS patients. **(E)** *I*–*V* before and after NTX stimulation in a cell as shown in **(D). (F)** *I*–*V* before and after PregS stimulation in a cell as shown in **(D). (G)** Bar graphs representing TRPM3 current amplitude at +100 mV after successive applications of 100 μM PregS in ME/CFS patients (*N* = *8*; *n* = 19 and *n* = 19) compared with HC (*N* = 8; *n* = 23 and *n* = 21). Data are represented as mean ± SEM. ME/CFS, myalgic encephalomyelitis/chronic fatigue syndrome; HC, healthy controls; NK, natural killer; PregS, Pregnenolone sulfate; TRPM3, Transient Receptor Potential Melastatin 3. ***p = 0.0006.

**Figure 7:**
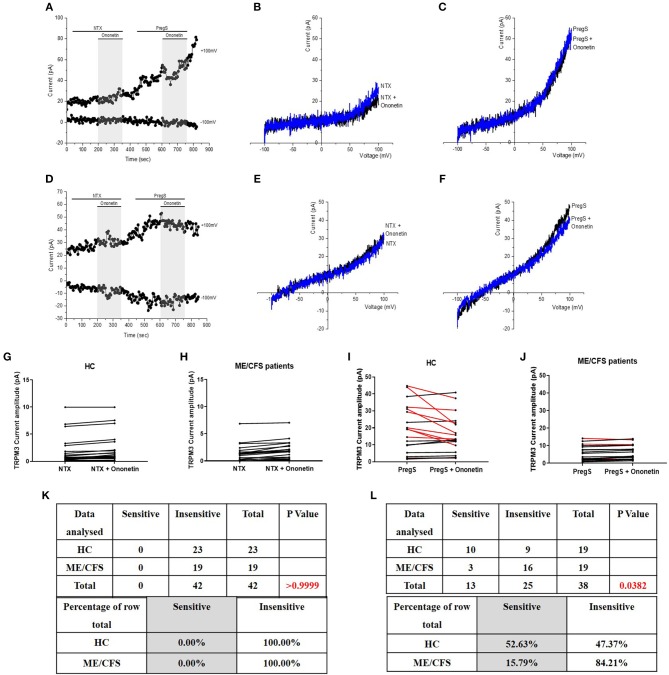
Modulation of NTX- and PregS- evoked currents with Ononetin. Data were obtained under whole-cell patch clamp conditions. **(A)** A representative time-series of current amplitude at +100 mV and −100 mV showing the effect of 10 μM ononetin (gray) on ionic currents in the presence of 200 μM NTX or 100 μM PregS in IL-2 stimulated NK cells from HC. **(B)** *I*–*V* before and after application of ononetin in the presence of NTX in a cell as shown in **(A). (C)** *I*–*V* before and after application of ononetin in the presence of PregS in a cell as shown in **(A). (D)** A representative time-series of current amplitude at +100 mV and −100 mV showing the effect of 10 μM ononetin (gray) on ionic currents in the presence of 200 μM NTX or 100 μM PregS in IL-2 stimulated NK cells ME/CFS patients. **(E)** *I*–*V* before and after application of ononetin in the presence of NTX in a cell as shown in **(D)**. **(F)** *I*–*V* before and after application of ononetin in the presence of PregS in a cell as shown in **(D). (G,H)** Scatter plots representing change of each current amplitude before and after application of ononetin in presence of NTX in all NK cells from HC and ME/CFS patients. Each cell represented as red lines had reduction in currents by ononetin. **(I,J)** Scatter plots representing change of each current amplitude before and after application of ononetin in presence of PregS in all NK cells from HC and ME/CFS patients. Each cell represented as red lines had reduction in currents by ononetin. **(K)** Table summarizing data for sensitive and insensitive cells to 10 μM ononetin in presence of NTX in HC (*N* = 8; *n* = 23) compared to ME/CFS patients (*N* = 8; *n* = 19). **(L)** Table summarizing data for sensitive and insensitive cells to 10 μM ononetin in presence of PregS in HC (*N* = 8; *n* = 19) compared to ME/CFS patients (*N* = 8; *n* = 19). Data are analyzed with Fisher's exact test. ME/CFS, myalgic encephalomyelitis/chronic fatigue syndrome; HC, healthy controls; NK, natural killer; NTX, naltrexone hydrochloride; PregS, Pregnenolone sulfate; TRPM3, Transient Receptor Potential Melastatin 3.

Lymphocyte populations were identified using forward and side scatter dot plots. Exclusions were CD3^+^ cells and only CD3^−^ lymphocytes were further used to characterize NK cell subset populations using CD56 and CD16. NK cell subsets were characterized using the surface expression of CD56^Bright^CD16^Dim/−^ NK cells and CD56^Dim^CD16^Bright/+^ NK cells. Cytometry data was exported from FacsDiva v8.1 and analyzed using SPSS v24 (IBM Corp, Version 24, Armonk, NY, USA) and GraphPad Prism v7 (GraphPad Software Inc., Version 7, La Jolla, CA, USA). Electrophysiological data were analyzed using pCLAMP 10.7 software (Molecular Devices, Sunnyvale, CA, USA). Origin 2018 (OriginLab Corporation, Northampton, MA, USA) and GraphPad Prism v7 (GraphPad Software Inc., Version 7, La Jolla, CA, USA) were used for statistical analysis and data presentation. Shapiro-Wilk normality tests were conducted to determine the distribution of data, in addition to skewness and kurtosis tests to determine data normality. Statistical comparison was performed using the independent non-parametric Mann-Whitney U test (Table 1, Figures 1, 2, **4**, **6**), and Fishers exact test (Figures 3, **5**, **7**), to determine any significant differences. Significance was set at *p* < 0.05 and the data are presented as mean ± SEM unless otherwise stated.

**Table 1:** Blood parameters and patient demographic.

	**HC**	**ME/CFS**	***P*-value**
Age (years)	34.75 ± 3.94	40.25 ± 4.66	0.442
Gender (%)	F (100%)	F (100%)	1.000
BMI (kg/m^2^)	23.39 ± 1.17	23.78 ± 1.42	1.000
WHODAS	2.96 ± 1.05	24.88 ± 4.31	0.000
**SF-36**			
General health (%)	78.64 ± 6.07	24.16 ± 5.31	0.000
Physical functioning (%)	92.50 ± 5.43	34.38 ± 5.63	0.000
Role physical (%)	79.38 ± 13.58	28.13 ± 11.21	0.038
Role emotional (%)	96.88 ± 3.13	79.16 ± 8.33	0.083
Social functioning (%)	92.19 ± 6.22	26.69 ± 4.05	0.003
Body pain (%)	87.81 ± 5.42	50.94 ± 7.90	0.000
**Pathology**			
White cell count (×10^9^/L)	5.51 ± 0.31	6.24 ± 0.30	0.130
Lymphocytes (×10^9^/L)	1.69 ± 0.13	2.03 ± 0.15	0.130
Neutrophils (×10^9^/L)	3.19 ± 3.15	3.60 ± 0.27	0.195
Monocytes (×10^9^/L)	0.4 ± 0.03	0.38 ± 0.03	0.645
Eosinophils (×10^9^/L)	0.16 ± 0.04	0.19 ± 0.05	0.645
Basophils (×10^9^/L)	0.05 ± 0.01	0.05 ± 0.01	0.645
Platelets (×10^9^/L)	239.25 ± 13.54	252.00 ± 16.27	0.505
Red cell count (×10^12^/L)	4.57 ± 0.06	4.36 ± 0.12	0.161
Haematocrit	0.41 ± 0.01	0.40 ± 0.01	0.505
Hemoglobin (g/L)	135.75 ± 2.45	133.50 ± 3.35	0.721

*SF-36 scores were analyzed using participant questionnaire responses. Results from routine full blood were analyzed in ME/CFS patients and HC. Data presented as mean ± SEM. ME/CFS, myalgic encephalomyelitis/chronic fatigue syndrome; HC, healthy controls; BMI, body mass index; WHODAS, World Health Organization Disability Assessment Schedule*.

## Results

### Participant Characteristics and Blood Parameters

Sixteen age- and sex-matched participants were included for this investigation. Demographic and clinical data for patients are summarized in Table 1. There was no significant difference in age or gender between patients and HC. The 36-Item Short Form Survey (SF-36) and World Health Organization Disability Assessment Schedule (WHODAS) were used to determine participant health-related-quality of life (45, 46). As expected, there was a significant difference in SF-36 and WHODAS scores between ME/CFS patients and HC. Moreover, full blood count parameters were measured for each healthy participant. All participant results were within the specified reference ranges for each parameter. There were no significant differences between healthy participants and ME/CFS patients for these reporting parameters as provided by the Gold Coast University Hospital Pathology Unit, NATA accredited laboratory.

### Natural Killer Cell Purity

NK cell purity (CD3^−^/CD56^+^) was 90.34% ± 0.6782 for HC and 90.9% ± 1.695 for ME/CFS patients as determined by flow cytometry (Figure 1A). There was no significant difference in NK cell purity in ME/CFS patients compared with HC (Figure 1B).

### Effects of Successive Applications of PregS on TRPM3 Activity

Electrophysiology recordings were obtained from isolated human NK cells incubated for 24 h with IL-2 using whole-cell patch-clamp technique. Endogenous TRPM3 channel activity was stimulated by two successive applications of 100 μM PregS enabling measurement of a small outwardly rectifying current under voltage-clamp conditions and observation of a typical shape of the TRPM3 current–voltage relationship (*I*–*V*) (Figure 2). Indeed, we measured a small ionic current with a typical TRPM3-like outward rectification in NK cells isolated from HC after both successive additions of PregS (Figures 2B,C). In contrast and as previously reported (28, 29), the outward ionic current amplitudes were significantly decreased after successive PregS stimulations in NK cells from ME/CFS patients in comparison to HC (Figures 2E–G) (*p* < 0.0001 and *p* = 0.0001). These results validate the previous findings and report that TRPM3 channel activity is impaired after successive PregS applications on IL-2 stimulated NK cells from ME/CFS patients.

### Modulation of PregS- Evoked Currents With Ononetin

Ononetin effectively inhibits PregS-induced Ca^2+^-influx and ionic currents through TRPM3 ion channels (47). Therefore, to confirm that TRPM3 activity is involved in ionic currents evoked by PregS in NK cells, we next used 10 μM ononetin to modulate the TRPM3 ion channels (Figure 3). As previously shown (28, 29), the ionic currents evoked by both successive applications of PregS were effectively inhibited by simultaneous application of ononetin in isolated NK cells from HC (Figures 3G,I,K,L). Moreover, the *I*–*V* of ononetin sensitive currents was outwardly-rectified and typical for TRPM3 (Figures 3B,C). In contrast, ionic currents evoked by both successive applications of PregS were mostly resistant to ononetin in isolated NK cells from ME/CFS patients (Figures 3E,F,H,J) in comparison with HC (Figures 3K,L) (*p* = 0.0030 and *p* = 0.0035). Collectively, these results confirm the significant loss of the TRPM3 channel activity in NK cells from ME/CFS patients after stimulation with IL-2 for 24 h.

### Effects of Successive Applications of PregS on TRPM3 Activity After Treatment With NTX

Stimulated IL-2 NK cells were co-treated with 200 μM NTX for 24 h as described previously (36). Indeed, at this concentration and incubation time, NTX has been reported to not affect cell viability or induce apoptosis of immune cells. As NTX acts as an antagonist to the μOR thus negating the inhibitory function of this opioid receptor on TRPM3, we tested whether the amplitudes of the PregS-induced currents were modified in NK cells treated with NTX from both HC and ME/CFS patients. Successive PregS applications (100 μM) evoked small ionic currents (Figures 4B,C) with a typical shape of the TRPM3 current–voltage relationship (*I*–*V*) in NK cells isolated from HC; however, no significant difference was reported within the HC group between NK cells treated or not with NTX (Figure 4H). In contrast, PregS applications in NTX-treated NK cells from ME/CFS patients mimicked the PregS-induced increase in NK cells from HC (Figures 4E–G,I). Indeed, successive PregS stimulations induced a significant increase of the outwardly rectified current amplitudes in NTX-treated NK cells from ME/CFS patients in comparison with non-treated NK cells from ME/CFS patients and to the same level as treated and non-treated NK cells from HC (Figures 4G–I) (*p* = 0.0001 and *p* = 0.0035). Moreover, the PregS-evoked currents present a typical shape of the TRPM3 current–voltage relationship (*I*–*V*) (Figures 4E,F). The data suggest that NTX restores TRPM3 channel activity in ME/CFS patients.

### Modulation of PregS- Evoked Currents With Ononetin After Treatment With NTX

To confirm that TRPM3 activity is involved in ionic currents evoked by PregS in NTX-treated NK cells from ME/CFS patients, we next used 10 μM ononetin to modulate the TRPM3 ion channels (Figure 5). As NTX did not modify the outwardly rectified current amplitudes in NTX-treated NK cells from HC, no difference was shown in HC and the ionic currents evoked by both successive applications of PregS were effectively inhibited by simultaneous application of ononetin (Figures 5G,I,K,L). In contrast, the ionic currents evoked by successive PregS applications were effectively inhibited by simultaneous application of 10 μM ononetin in isolated NTX-treated NK cells from ME/CFS patients (Figures 5E,F,H,J–L). Indeed, the ionic current initially resistant to ononetin in isolated NK cells from ME/CFS patients became sensitive to ononetin after incubation with NTX for 24 h (Figure 5L). Moreover, the *I*–*V* of ononetin sensitive currents was outwardly-rectified and typical for TRPM3 (Figures 5E,F). Collectively, these results confirm the restoration of TRPM3 channel activity in NK cells from ME/CFS patients after treatment with NTX for 24 h.

### Effects of Successive Applications of NTX and PregS on TRPM3 Activity

Opioid antagonists, such as NTX, have been previously reported to not affect TRPM3-dependent Ca^2+^ signals when directly applied in perfusion (33). Therefore, to verify whether NTX can directly regulate TRPM3 channel activity, we stimulated TRPM3 in NK cells using only the opioid antagonist NTX (Figure 6). No TRPM3-like ionic currents were induced by NTX (200 μM). Indeed, we did not measure any outwardly rectifying currents under voltage-clamp conditions with a typical shape of the TRPM3 current–voltage relationship (*I*–*V*) in IL-2 stimulated NK cells from either HC (Figures 6B,G) or ME/CFS patients (Figures 6E,G) after addition of NTX. However, as PregS is currently the most potent TRPM3 agonist described in the literature (48) and to confirm that TRPM3 ion channel activity could be stimulated, we then applied 100 μM PregS to the NK cells from both HC and ME/CFS patients (Figures 6A,B). Application of PregS induced TRPM3-like currents in both HC (Figures 6C,G) and ME/CFS patients (Figures 6F,G). The *I-V* of the PregS-evoked currents was outwardly rectified (Figures 6C,F), which is standard for TRPM3. However, the outward current amplitudes decreased significantly after PregS stimulation in NK cells from ME/CFS patients (Figure 6G) (*p* = 0.0006) as previously shown (Figure 4G). The data suggest that TRPM3 channel activity is not directly stimulated by NTX in either HC or ME/CFS patients. Therefore, NTX is not a direct agonist of TRPM3 ion channel.

### Modulation of NTX- and PregS- Evoked Currents With Ononetin

As no TRPM3-like ionic currents were induced after stimulation with NTX, no difference was reported with simultaneous application of 10 μM ononetin in either IL-2 stimulated NK cells from HC (Figures 7B,G,K) or ME/CFS patients (Figures 7E,H,K). In contrast, the ionic currents evoked by PregS were effectively inhibited by simultaneous application of 10 μM ononetin in isolated NK cells from HC (Figures 7C,I,L). In addition, ionic currents in the presence of PregS were mostly resistant to ononetin in isolated NK cells from ME/CFS patients (Figures 7F,J,L), in comparison with HC, confirming that TRPM3 channel activity is not directly modulated by NTX compared with PregS.

## Discussion

Our previous studies have described the loss of TRPM3 ion channel function to be associated with ME/CFS (28, 29). In the present study, we used whole-cell patch-clamp technique as a method to measure endogenous TRPM3 activity in IL-2 stimulated NK cells from HC and ME/CFS patients, enabling the ion channel current recordings under voltage-clamp conditions and observation of the shape of the TRPM3 current–voltage relationship (*I*–*V*). This novel study confirms the significant loss of the TRPM3 channel activity after modulation with PregS and ononetin on a new cohort of ME/CFS participants (Figures 2, 3). Additionally, we report a similar effect after a second modulation with PregS and ononetin on IL-2 stimulated NK cells from ME/CFS patients. Indeed, successive application of PregS enabled the measurement of small ionic currents with a typical TRPM3-like outward rectification in IL-2 stimulated NK cells isolated from HC. In contrast, the amplitude of outward ionic currents decreased significantly after successive PregS-stimulation in IL-2 stimulated NK cells from ME/CFS patients, confirming impaired TRPM3 channel activity in ME/CFS patients. In addition, PregS-evoked ionic currents through TRPM3 ion channels were significantly modulated by ononetin in IL-2 stimulated NK cells from HC compared with ME/CFS patients. Indeed, ionic currents in the presence of PregS were mostly resistant to ononetin in IL-2 stimulated NK cells from ME/CFS patients.

We now report, for the first time, that TRPM3 channel activity was restored in IL-2 stimulated NK cells isolated from ME/CFS patients after incubation with 200 μM NTX for 24 h (Figures 4, 5). We demonstrated that the treatment with NTX for 24 h had no effect on the PregS-evoked TRPM3-like currents in HC, whereas the outward current amplitudes increased significantly after successive PregS stimulations in ME/CFS patients. In addition, treatment with NTX also restored the sensitivity of NK cells from ME/CFS patients to ononetin, confirming that TRPM3 activity was involved in ionic currents evoked by PregS in NTX-treated NK cells from ME/CFS patients. Finally, we report that direct application of NTX does not affect TRPM3-dependent Ca^2+^ signals when tested before PregS stimulation on both IL-2 stimulated NK cells from HC and ME/CFS patients (Figures 6, 7), suggesting that NTX does not act as an agonist by directly coupling on the TRPM3 ion channel gating but may involve an upstream lasting regulatory mechanism.

TRPM3 ion channels act as integrators of several stimuli and signaling pathways. TRPM3, expressed abundantly in sensory neurons, are defined as thermosensitive and nociceptor channels that help organisms to sense heat and make them more sensitive to pain during inflammation (27). Dysregulation of thermoregulatory responses has been reported in ME/CFS patients and generalized pain as well as brain inflammation are characteristics of ME/CFS (49). Physiologically, endogenous opioids are synthesized *in vivo* to modulate pain mechanisms and inflammatory pathways. Endogenous and exogenous opioids mediate analgesia in response to painful stimuli binding to opioid receptors, members of the family of GPCRs, on neuronal cells (38). Interestingly, molecules that bind to and activate opioid receptors significantly reduce TRPM3-dependent pain. Recent reports suggest that the activation of TRPM3 channels is greatly reduced if the channels are also stimulated by any of a variety of GPCRs, and particularly by the μOR responsible for analgesic, rewarding and unwanted effects of opioids (33, 34). Under activation by opioids—whether produced by the body (endogenously) or taken as a drug—μOR can mediate a spectrum of acute signaling and longer-term regulatory behaviors (50). Such functional versatility cannot be explained by a simple “on-off” switch model of receptor activation and is more compatible with dynamic and flexible receptor structures. Indeed, μOR interact with, and activate heterotrimeric G proteins to amplify the signal and regulate downstream effectors (51).

Recent studies have reported that TRPM3 inhibition requires the heterotrimeric G protein to dissociate in order to prevent the inhibitory G_α*i*_ subunit from interacting with the activated μOR, locking the complex in the resting trimeric state (33–35). On the other hand, it has been demonstrated that TRPM3 activity is strongly inhibited by the direct interaction with G_βγ_ as the flow of ions through TRPM3 channels is strongly and reversibly inhibited when the cell membrane is flushed with purified G_βγ_ (33, 35). Interestingly, TRPM3 channels require Phosphatidylinositol 4,5-bisphosphate (PIP_2_) in the membrane in order to open, and some other G_α_ subunits enhance the inhibition of TRPM3, by acting on the localized depletion of PIP_2_ (35). Therefore, specific assembly of μOR—G proteins complexes regulate the opening and closing of the TRPM3 ion channel pore and in turn impact on the Ca^2+^ signaling cascade and biological responses.

Importantly, opioid receptors are widely distributed on tissues and organ systems outside the CNS, such as the cells of the immune system, indicating that opioids are capable of exerting additional effects in the periphery, such as immunomodulatory and immunosuppressive effects (38). Evidence now exists showing that the analgesic effects of opioids are not exclusively mediated by opioid receptors in the brain but can also be mediated via the activation of receptors in immune cells. In turn this mechanism results in different side effects including immune suppressive functions and exacerbation of certain disease states (38). Although opioids modulate both innate and adaptive immune systems, defects in innate immunity appear to have broader consequences. For example, opioids act as immunosuppressors on migration and functional activity of innate immune responders by modulating leukocyte recruitment, cytokine secretion and bacterial clearance leading to impairment of the hosts' ability to eradicate pathogens (38). Interestingly, immune-derived opioid peptides also play a substantial role in the modulation of inflammatory pain. For example, (β)-endorphin (βEP), an endogenous opioid neuropeptide and peptide hormone, has been reported to be produced by T- and B-lymphocytes as well as in monocytes, in inflamed rat paw (52). βEP acts primarily on μOR and δOR and modulates the functions of lymphocytes and other cells involved in host defense and immunity (53). Recently, NTX has been shown to promote δOR activity and enhance NK cell cytolytic activity in response to βEP *in vitro* on rat splenocytes, suggesting the potential use of NTX in the treatment of immune deficiency (54). The δOR agonistic activity of NTX on splenocytes appears to be due to its potent μOR antagonistic function as δOR expression is tightly controlled by a negative feedback regulation of μOR. NTX disrupts this feedback control by reducing μOR function, thereby upregulating δOR binding that results in enhanced NK cell cytolytic response to the ligands (54). Interestingly, an investigation by Conti et al. reported significantly reduced βEP in ME/CFS patients reflecting the condition of chronic immune activation (55).

Importantly, NTX acts as an antagonist to the μOR thus negating the inhibitory function of this opioid receptor on TRPM3 (37, 38). Indeed, opioids binding to μOR block Ca^2+^ influx through inhibition of TRPM3 channels. The disinhibiting effect of NTX restores the TRPM3 ion channel activity in ME/CFS patients and in turn re-establishes the appropriate Ca^2+^ signaling. Each cell type, including NK cells, has a unique signaling phenotype capable of delivering Ca^2+^ signals with the spatial and temporal properties necessary to regulate its particular function (24). Phenotypic stability seems to be maintained by Ca^2+^- dependent feedback mechanisms that adjust the expression levels of individual toolkit components that contribute to these cell-specific signaling systems. These homoeostatic systems are highly plastic and can undergo a process of phenotypic remodeling, resulting in the Ca^2+^ signals being set either too high or too low. Such subtle dysregulation of Ca^2+^ signals may highly impact cell functions and result in diseases (24, 56). Release of Ca^2+^ by TRPM3 seems to play a central role in NK cells' activation and function as dysregulation of TRPM3 function in ME/CFS patients has been shown to affect Ca^2+^ signaling, leading to impaired NK cell regulatory machinery and functions. Restoration of TRPM3 channel activity by NTX leads to Ca^2+^ signal remodeling that will in turn contribute to cells' activation, effector functions, gene expression or differentiation. Thus, restoring Ca^2+^ homeostasis in NK cells will help to reactivate and rebalance the different Ca^2+^ dependent mechanisms including ongoing transcriptional processes, modulation of TRPM3 surface expression, or constitutive and regulated vesicular trafficking of the ion channel itself or of accessory and regulating proteins. Hence restoration of Ca^2+^ homeostasis results also in the restoration of the integrity and stability of these NK cell-specific signaling systems.

In conclusion, ME/CFS is a long-term illness with a wide range of symptoms, including chronic pain, impaired memory and concentration, and inflammation (1). The widely expressed Ca^2+^ permeable nonselective cation channel, TRPM3, functions as a chemo- and thermo-sensor enabling the detection of painful and innocuous thermal stimuli (27). TRPM3 dysfunction has been reported to contribute to the pathological state of ME/CFS (28–32). In the present study, we confirmed impaired TRPM3 activity in ME/CFS patients through electrophysiological investigations in IL-2 stimulated NK cells after both successive activation with PregS and inhibition with ononetin. Importantly, we demonstrated that TRPM3 channel activity was restored in IL-2 stimulated NK cells isolated from ME/CFS patients after incubation with NTX for 24 h. Indeed, the opioid antagonist NTX negates the inhibitory function of opioid receptors on TRPM3 in NK cells from ME/CFS patients. This study not only helps to provide an understanding of the etiology and pathomechanism of ME/CFS, but confirms that opioid receptors in immune cells have an important role in analgesic effects. Finally, the restoration of TRPM3 channel activity suggests an immune-modulating action of NTX by remodeling the TRPM3-dependent Ca^2+^ signals, which will in turn affect NK cell functions, and support the hypothesis that NTX may have potential for use as a treatment for ME/CFS.

## Data Availability Statement

All datasets generated for this study are included in the manuscript/supplementary files.

## Ethics Statement

All participants gave written consent to participate and to publish. Ethics approval was under Human Research Ethics Committee Griffith University (HREC/15/QGC/63).

## Author Contributions

HC, KM, SM-G, and DS designed the study and wrote the manuscript. HC performed experiments. HC and KM performed data analysis.

### Conflict of Interest

The authors declare that the research was conducted in the absence of any commercial or financial relationships that could be construed as a potential conflict of interest.

## Abbreviations

7-AAD: 7-amino-actinomycin
βEP: Beta endorphins
δOR: Delta opioid receptor
μOR: Mu opioid receptor
BMI: Body Mass Index
Ca^2+^: Calcium
[Ca^2+^]_i_: Intracellular Ca^2+^ concentration
CCC: Canadian Consensus Criteria
CDC: Centers for Disease Control and Prevention
CNS: Central nervous system
EDTA: Ethylendiaminetetraacetic acid
FBS: Fetal bovine serum
HC: Healthy controls
ICC: International Consensus Criteria
IL-2: Interleukin-2
IL-12: Interleukin-12
*I-V*: Current–voltage relationship
ME/CFS: Myalgic Encephalomyelitis/Chronic Fatigue Syndrome
NCNED: National Centre for Neuroimmunology and Emerging Diseases
NK: Natural killer
NTX: Naltrexone hydrochloride
PBMCs: Peripheral blood mononuclear cells
PIP_2_, Phosphatidylinositol 4: 5-bisphosphate
PregS: Pregnenolone sulfate
RPMI: Roswell Park Memorial Institute medium
SF-36: 36-Item Short Form Survey
SNPs: Single nucleotide polymorphisms
TRP: Transient receptor potential
TRPM: Transient receptor potential melastatin
TRPM3: Transient receptor potential melastatin 3
WHODAS: World Health Organization Disability Assessment Schedule.
